# Eflornithine for chemoprevention in the high-risk population of colorectal cancer: a systematic review and meta-analysis with trial sequential analysis

**DOI:** 10.3389/fonc.2023.1281844

**Published:** 2023-11-15

**Authors:** Lifeng Yang, Yan Wang, Shasha Hu, Xiaoyan Wang

**Affiliations:** ^1^ School of Nursing, Hexi University, Zhangye, China; ^2^ Peking University First Hospital Ningxia Women and Children’s Hospital (Ningxia Hui Autonomous Region Maternal and Child Health Hospital), Nursing Department, Yinchuan, Ningxia Hui Autonomous Region, China; ^3^ The First Ward of the Department of Gynecology, The First Hospital of Lanzhou University, Lanzhou, China

**Keywords:** Eflornithine, colorectal cancer, high-risk group, chemoprevention, meta-analysis, trial sequential analysis

## Abstract

**Objectives:**

To evaluate the efficacy of Difluoromethylornithine (DFMO) chemoprevention in the high-risk population for colorectal cancer (CRC).

**Methods:**

Meta-analysis was conducted to assess the caliber of the included literature by searching five databases for randomized controlled trials of DFMO chemoprevention in the high-risk population of CRC, with RevMan 5.4, Stata 15.0 and TSA 0.9.5.10 employed to statistically analyze the extracted data. Grade profiler 3.6 was employed for grading the evidence for the outcome indicators (disease progression and adenoma incidence).

**Results:**

Six trials were finally included in this research, with the collective data indicating that the DFMO combination therapy was efficacious in lowering the incidence of recurrent adenomas in patients who had experienced advanced CRC [RR 0.34, 95% CI 0.14 - 0.83, P < 0.05]. Meta-analysis showed that DFMO combined therapy had no statistical difference in disease progression in patients with familial adenomatous polyposis[RR 0.52, 95% CI 0.14 - 1.86, P > 0.05]; Trial Sequential Analysis reveals that the combination therapy of DFMO effectively diminishes the occurrence of recurrent adenomas in patients with a history of advanced colorectal tumors, displaying a Risk Ratio (RR) of 0.33 with a 95% Confidence Interval (CI) of 0.12 - 0.90 and a significance level of P < 0.05. This combination exhibits a statistically significant difference. Subgroup analysis demonstrates that, depending on the drug treatment regimen (DFMO+ Aspirin/DFMO+ Sulindac), the combination of DFMO and aspirin exhibits an effect comparable to a placebo in diminishing the occurrence of new adenomas in patients with a history of advanced colorectal tumors. However, the combination of DFMO and sulindac significantly mitigates the incidence of recurrent adenomas in this patient population.

**Conclusion:**

This meta-analysis indicates that the existing randomized controlled trials are adequate to ascertain the efficacy of DFMO combination therapy in diminishing the incidence of recurrent adenomas in patients who have previously encountered advanced colorectal tumors. However, further clinical trials need to be conducted to evaluate the optimum dosage and treatment course of prophylactic implementation of DFMO combination therapy in high-risk populations.

## 1 Introduction

Colorectal cancer (CRC) is a highly prevalent and fatal disease worldwide (1, 2). Globally, CRC causes approximately 1.8 million new cases with 900,000 deaths reported each year. It ranks as the third most prevalent malignancy and the second most common cause of cancer death, estimated by the International Agency for Research on Cancer (IARC) in 2018 (3). Most CRCs develop from adenomas, which, if left untreated, carry a lifetime risk of CRC of up to 100% (4, 5), whereas removal of adenomas can reduce the risk of CRC. Although the incidence of CRC can be reduced by prophylactic resection of adenomatous polyps found at screening, chemoprophylactic therapy is gaining attention from clinicians because of its characteristic of low toxicity, cost-effectiveness, and high efficiency.

Chemopreventive drugs enhance the management of colorectal adenoma by delaying or avoiding the development of advanced colorectal adenomas. Additionally, the ideal chemopreventive drug should have a biologically plausible mechanism of action, be safe and easily tolerated over a longer treatment period, and produce long-lasting and clinically significant effects (6). Among the latest chemopreventive agents for CRC, difluoromethylornithine (DFMO) is considered to be the most promising agent. DFMO is an irreversible inhibitor of ornithine decarboxylase(ODC) (7), and it possesses cyto-inhibitory properties. Importantly, its threshold concentration for toxicity is also much higher than that required to inhibit ODC activity (8), and it does not inhibit any other polyamine-metabolizing enzymes. Therefore, DFMO has received widespread attention for its advantages such as low toxicity and significant preventive effects on certain tumors. This effect was noted especially in solid tumors of epithelial origin, with DFMO being included in several clinical trials for the prevention and treatment of malignancies.

Recently, several studies on the efficacy of DFMO chemoprevention for CRC have been published, but conflicting results were noted (9–14). In 2022, a report of a network meta-analysis involving 13 chemistries for the prevention of colorectal adenoma incidence (15) reported that DFMO combined with sulindac was optimally effective for the chemoprevention of colorectal adenoma. However, the study included only one randomized controlled trial (RCT), and the included number of samples was quite small. In addition, the results of studies on the efficacy of DFMO alone in treating patients with CRC are lacking, and the impact on a high-risk population with CRC needs further clarification. Hence, the primary objective of this meta-analysis is to evaluate the influence of DFMO on the recurrence of adenomas in populations at elevated risk for colorectal cancer. The secondary objective is to assess the advancements in research pertaining to the disease and to conduct subgroup analyses based on the following predefined drug treatment regimens (DFMO+ Aspirin/DFMO+ Sulindac).

## 2 Methods

### 2.1 Search strategy

This study was systematically reviewed, meta-analyzed, and reported according to Preferred Reporting Items for Systematic Reviews and Meta-Analyses (PRISMA) (16). Up to July 2023, PubMed, Embase, Web of Science, OVID, and the Cochrane Library were assessed for relevant research on DFMO for chemoprevention in the high-risk population of CRC. The study utilized subject terms combined with free words as search criteria (MeSH in PubMed and Emtree in Embase) using Population, Intervention, Comparison, Outcomes, and Study (PICOS) principles. There was no restriction on the language of publication. References from relevant reviews and meta-analyses were also reviewed to recognize eligible studies for this study. The strategy utilized for this particular search is described in Appendix 1. Moreover, the manual search was performed complementally in this study to discover pertinent literature.

### 2.2 Study selection

The inclusion criteria were: (a) study population: the population with increased risk of CRC (17) which includes familial adenomatous polyposis, previous history of colorectal adenomatosis, history of CRC, associated inflammatory bowel disease such as Crohn’s disease or ulcerative colitis; (b) intervention method: use of DFMO in combination with other drugs or DFMO alone for the treatment of colorectal disease; (c) control group: use of placebo or other similar drugs. (d) Main outcome: Incidence of recurrent colorectal adenomas (advanced adenomas and any adenomas); Secondary outcome: disease progression. Disease progression was defined as requiring surgery, advanced adenomas requiring endoscopic resection, the occurrence of high dysplasia, or stage progression of duodenal polyposis (10); (e) Study design: RCT study.

The exclusion criteria were: (a) review, individual cases, commentary, or case report; (b) animal experiments; (c) incomplete reported data; (d) duplicate publications or single articles published in multiple languages, and duplicate data publication (overlapping samples from several studies), the most informative articles were included to circumvent duplication of data.

### 2.3 Data collection and quality assessment

Two authors (WY and YLF) independently conducted study selection based on predetermined inclusion and exclusion criteria. Any Disagreements were resolved by thorough discussion. WY and WXY independently collected data from the included trials. The following information was extracted from each included article: first author, year of publication, region, drug type, sample size, dose, and outcome indicators. The authors of the included studies were contacted for additional information when needed.

This study evaluated the risk of bias according to the guidelines outlined in the Cochrane Handbook for the Evaluation of Intervention Systems (5.1.0) (18). HSS and WXY scrutinized all studies and designated “high,” “low,” or “unclear” values to the following categories: random sequence generation; allocation concealment, blinding of participant and investigator; blinding of outcome measures; incomplete outcome data; selective reporting; and other biases.

### 2.4 Statistical analysis

Relative risk ratios (RRs) were calculated for dichotomous outcomes at a 95% confidence interval (CI). A random-effects model was utilized for the meta-analysis of clinical heterogeneity. All analyses were conducted on an intention-to-treat basis. Heterogeneity was reported using I^2^ statistics, with I^2^ > 50% indicating significant heterogeneity (19).

Subgroup analyses were executed based on predefined drug treatment regimens (DFMO+ Aspirin/DFMO+ Sulindac), with sensitivity analyses conducted for all outcomes.

Egger’s test was used to evaluate publication bias (20), and *P* < 0.05 were considered a statistically significant difference. All statistical analyses were conducted employing Stata 15.0 (Stata Corporation, TX, USA) and RevMan 5.4 (Nordic Cochrane Centre, Denmark).

### 2.5 Trial sequential analysis

To address the primary outcomes, Trial Sequential Analysis (TSA) was undertaken utilizing TSA software. This approach mitigates the risks associated with inadequate sample sizes and successive updates of studies incorporated into the meta-analysis, which could potentially escalate the probability of random errors (21). The meta-analysis adjusted the Required Information Size (RIS) and computed the trial sequential monitoring boundaries to ascertain the reliability of the evidence within our meta-analysis (22).

### 2.6 Grading the quality of evidence

GRADE (23) was used to assess the overall quality of the evidence and the strength of the recommendations. The standard classifies evidence quality into four categories: very low, low, medium and high.

## 3 Results

### 3.1 Trial selection

**Figure 1:**
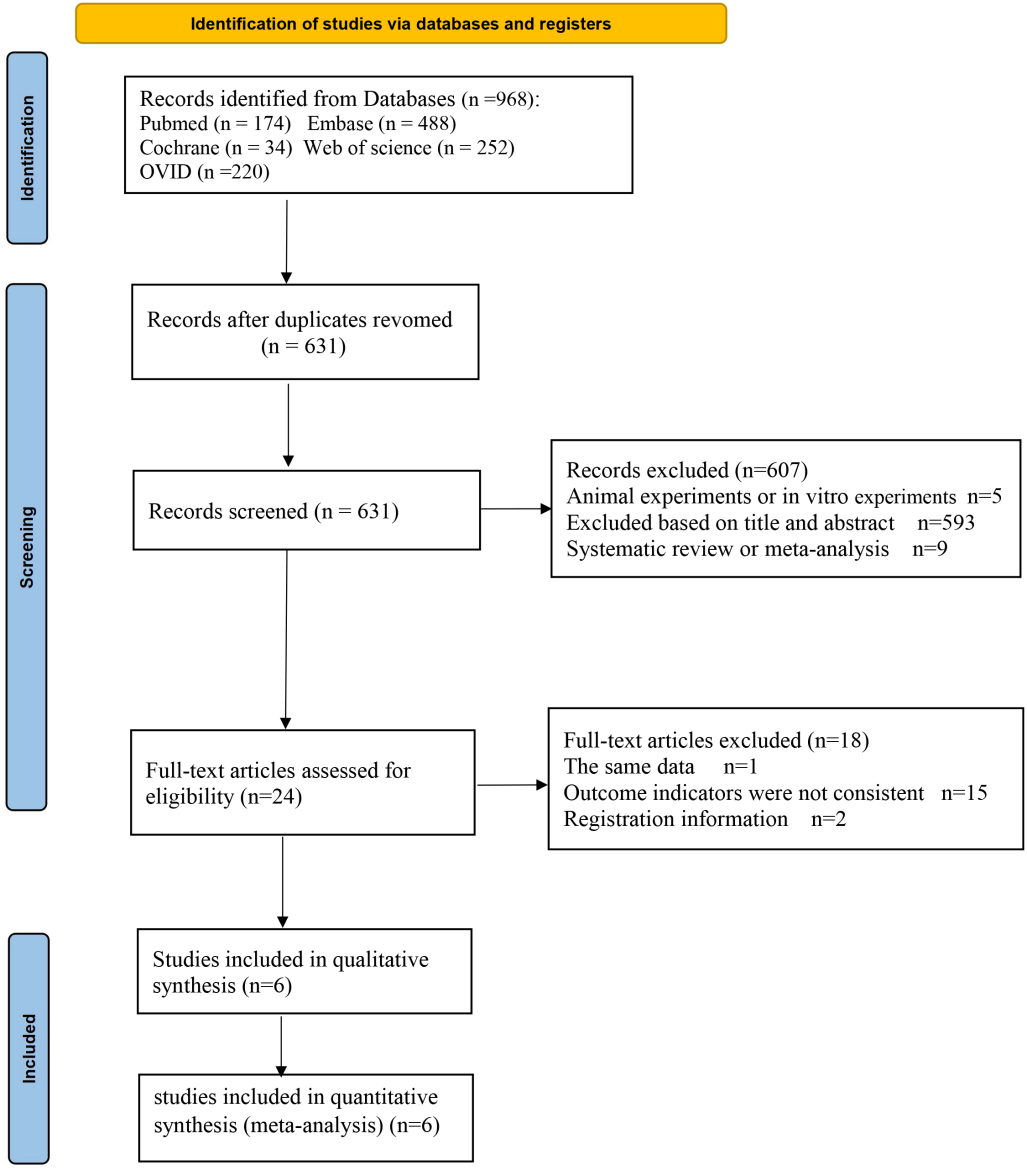
Flow diagram of the study selection process for the meta-analysis.

A total of 968 articles were identified from the online database. After removing duplicates, 631 articles remained for further consideration based on their titles and abstracts. The 593 articles that were not relevant to the topic were excluded, nine review articles and five animal experiments were eliminated as well, with 24 articles were being selected for full-text review. Among these, one article was excluded due to duplication of data, 2 articles are registration information, and 15 articles were excluded because the outcome indicators were not met. Six trials (9–14) were finally included (**Figure 1**).

### 3.2 Trial characteristics

The primary features of the six included RCTs are demonstrated in **Table 1**. These studies were published between 2008 and 2022. Among the six included studies, five were carried out in the United States (10–14) and one in Spain (9). Five RCTs used multicenter studies (9, 10, 12–14). Five RCTs used Eflornithine + Sulindac as treatment regimen (9, 10, 12–14), one RCT used Eflornithine + Aspirin as treatment regimen (11). Furthermore, two studies used Sulindac as a control (9, 10), and four studies used placebo as a control (11–14). Sample sizes for the six RCTs ranged from 86 to 375. In two studies the treatment period was two years (9, 10), in one study, the treatment period was one year (11), and in three studies, treatment period was three years (12–14). Four trials reported the incidence of adenomas in patients with advanced colorectal neoplasms (11–14), and two studies reported on the outcome of the progression of disease in patients with familial adenomatous polyposis (9, 10). One study (10) reported that the research was funded by the manufacturer.

**Table 1:** Characteristics of the included trials.

Study	Country	Setting	Sponsored by manufacturer	Population	Drug regimen(T/C)	Sample size(T/C)	Dosage(T/C)	Times	Outcomes
Francesc B. 2022 (9)	Spain	multi-center	/	Familial adenomatous polyposis	DFMO+Sulindac/Sulindac	54/53	750mg+150mg/150mg	48months	①
Burke, C. A. 2020 (10)	America	multi-center	Cancer Prevention Pharmaceuticals	Familial adenomatous polyposis	DFMO+Sulindac/Sulindac	56/58	750mg+150mg/150mg	48 months	①
Sinicrope, F. A. 2019 (11)	America	single-center	/	Prior advanced colorectal neoplasms	DFMO+ Aspirin/Placebo	42/44	500mg+325mg/N	24 months	②
Raj, K. P. 2013 (12)	America	multi-center	/	History of colorectal adenoma	DFMO+ Sulindac/Placebo	69/72	500mg+150mg/N	36 months	②
Zell, J. A. 2012 (13)	America	multi-center	/	History of colorectal adenoma	DFMO+ Sulindac/Placebo	43/43	N	36 months	②
Meyskens, F.L. 2008 (14)	America	multi-center	/	History of colorectal adenoma	DFMO+ Sulindac/Placebo	191/173	500mg+150mg/N	36 months	②

T, Treatment; C, Control; ①Disease progression, ②Detection rate of adenoma.

### 3.3 Risk bias evaluation

**Figure 2:**
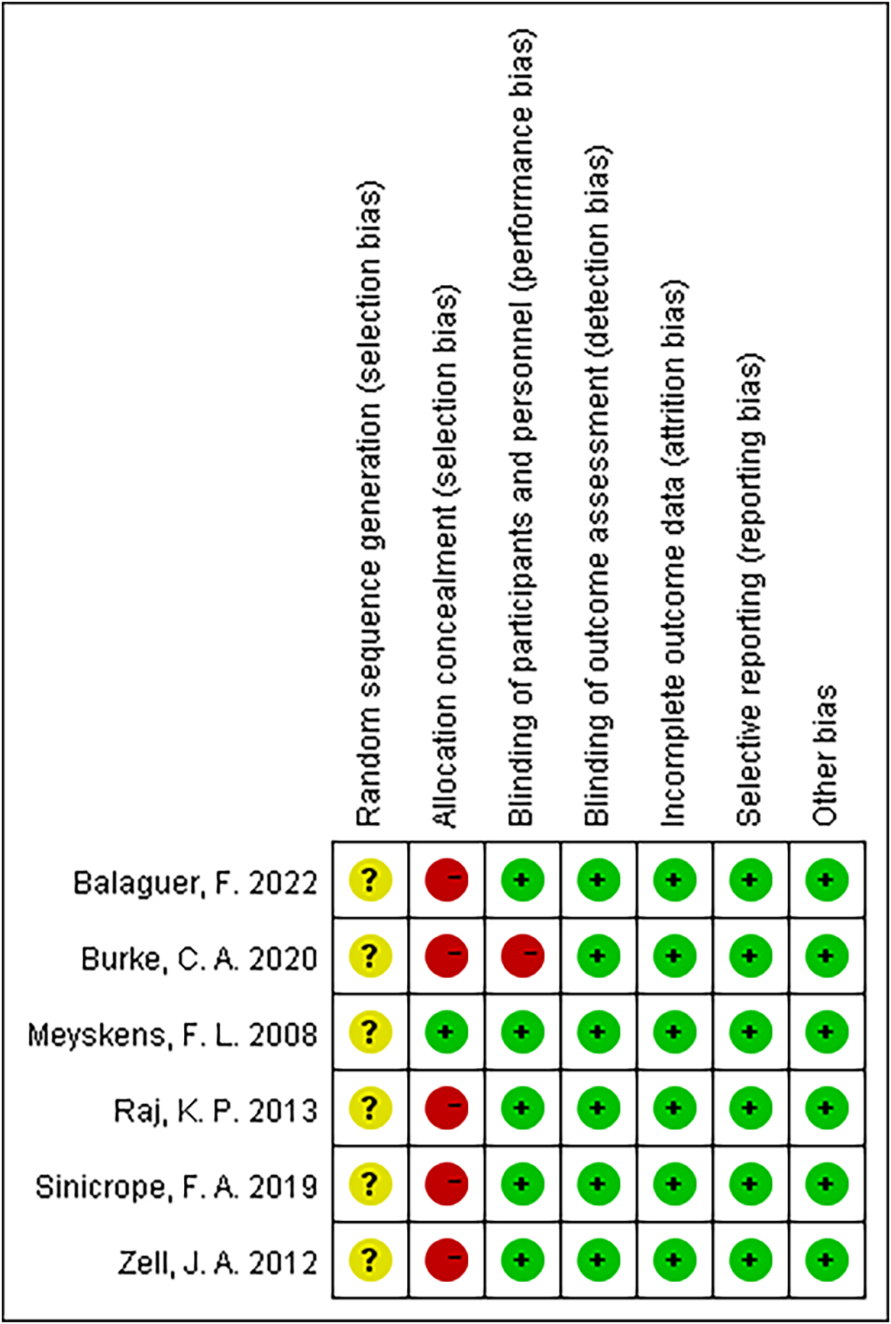
Risk of bias in included trials.

**Figure 2** summarizes the details of the risk of bias assessment. Six trials produced unclear randomized sequences (9–14), stating that only randomization was used without elaborating on the specific manner. One study used correct allocation concealment (14), and five studies did not mention random allocation concealment (9–13). Five studies were double-blinded (9, 11–14), and one study did not mention the use of the blinding method (10).

### 3.4 Outcome

#### 3.4.1 Disease progression

**Figure 3:**
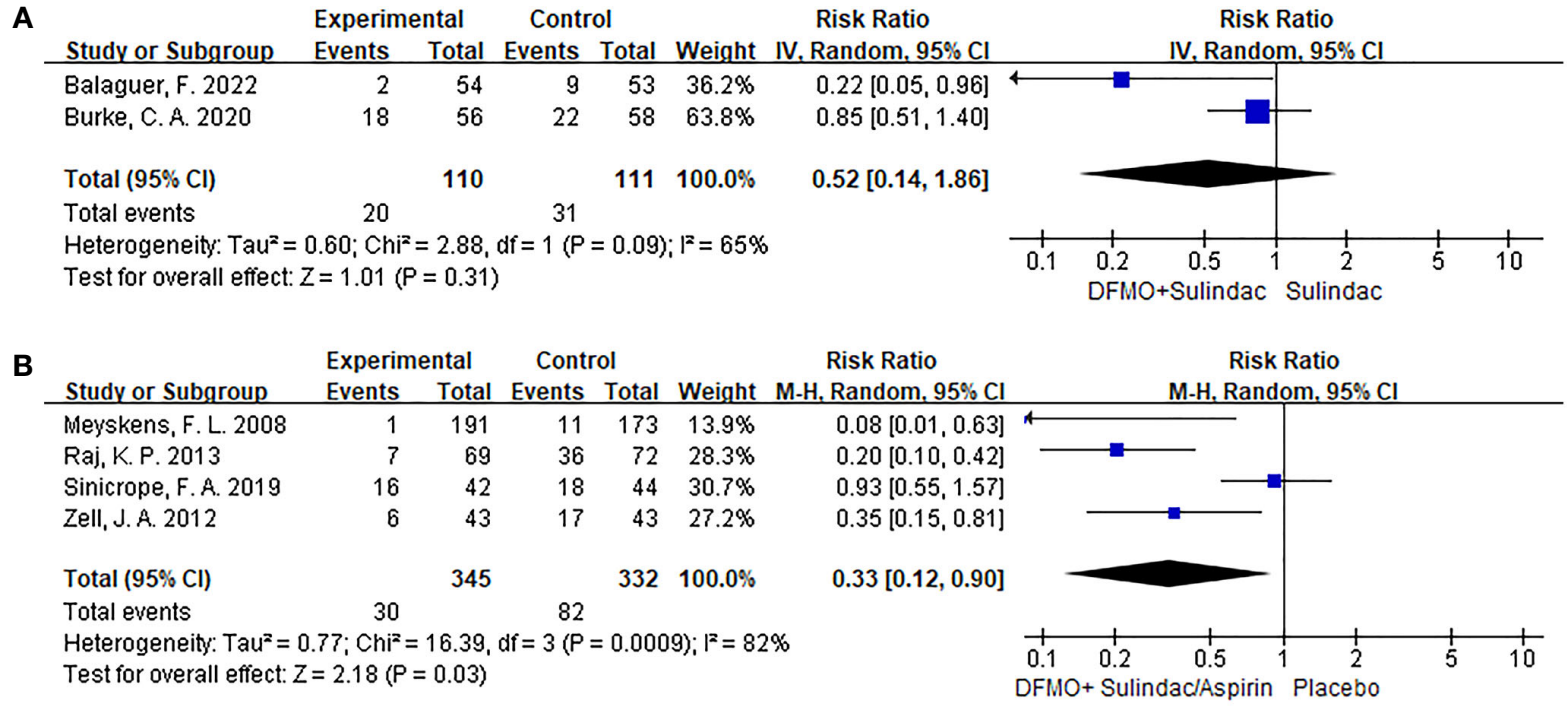
Forest plots of studies included comparing the disease progression and the detection recurrent rate of adenoma. A random-effects model was utilized for the meta-analysis of clinical heterogeneity. **(A)** Forest plot for disease progression in patients with colorectal cancer compared with DFMO combination therapy and sulindac; **(B)** Forest plot for the detection rate of recurrent adenoma in patients with colorectal cancer compared with DFMO combination therapy and placebo.

Two trials, including 221 participants, reported the effect of DFMO combination therapy on the progression of disease in patients with familial adenomatous polyposis. The data indicated that DFMO combination therapy had no impact on the reduction of disease progression in such patients relative to the control sulindac (RR 0.52, 95% CI 0.14 - 1.86, *P* > 0.05; I^2^ = 65%; **Figure 3A**). The sensitivity analysis showed no change in results after changing the effect model, indicating robust results.

#### 3.4.2 Incidence of recurrent colorectal adenomas (advanced adenomas and any adenomas)

In the analysis, four trials involving a total of 677 participants provided data on the impact of DFMO combinations on the incidence of recurrent adenomas in individuals who had previously experienced CRC. The DFMO combination therapy significantly reduced the incidence of recurrent adenomas in patients with previously advanced CRC in comparison to the control placebo group (RR 0.33, 95% CI 0.12 - 0.90, P < 0.05; I^2^ = 82%; **Figure 3B**). Sensitivity analysis exhibited no variance in the results after changing the effect model, which reflects the robustness of the results.

### 3.5 Subgroup analysis

**Figure 4:**
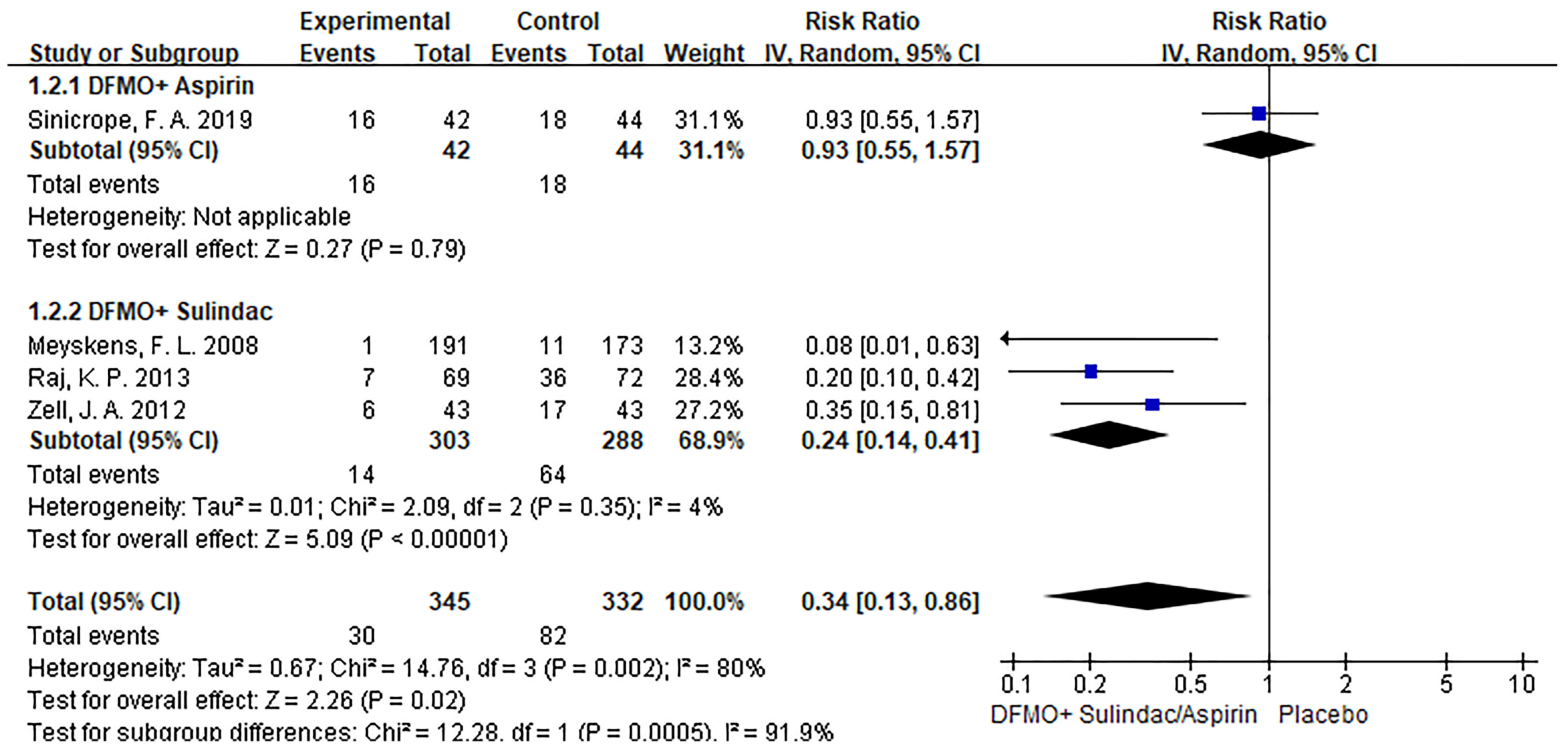
Subgroup analysis of detection rate of colorectal cancer adenoma with DFMO combined with drug therapy (DFMO+Aspirin/DFMO+ sulindac).

Subgroup analysis revealed that DFMO combined with aspirin was comparable to placebo in the incidence of recurrent adenomas in patients with previous advanced CRC. Nevertheless, DFMO combined with sulindac significantly reduced the incidence of new adenomas in patients with previous advanced CRC. Sensitivity analysis showed no change in the results after changing the effect model, which reflects the robustness of the results. (Show in **Figure 4**)

### 3.6 Publication bias

**Figure 5:**
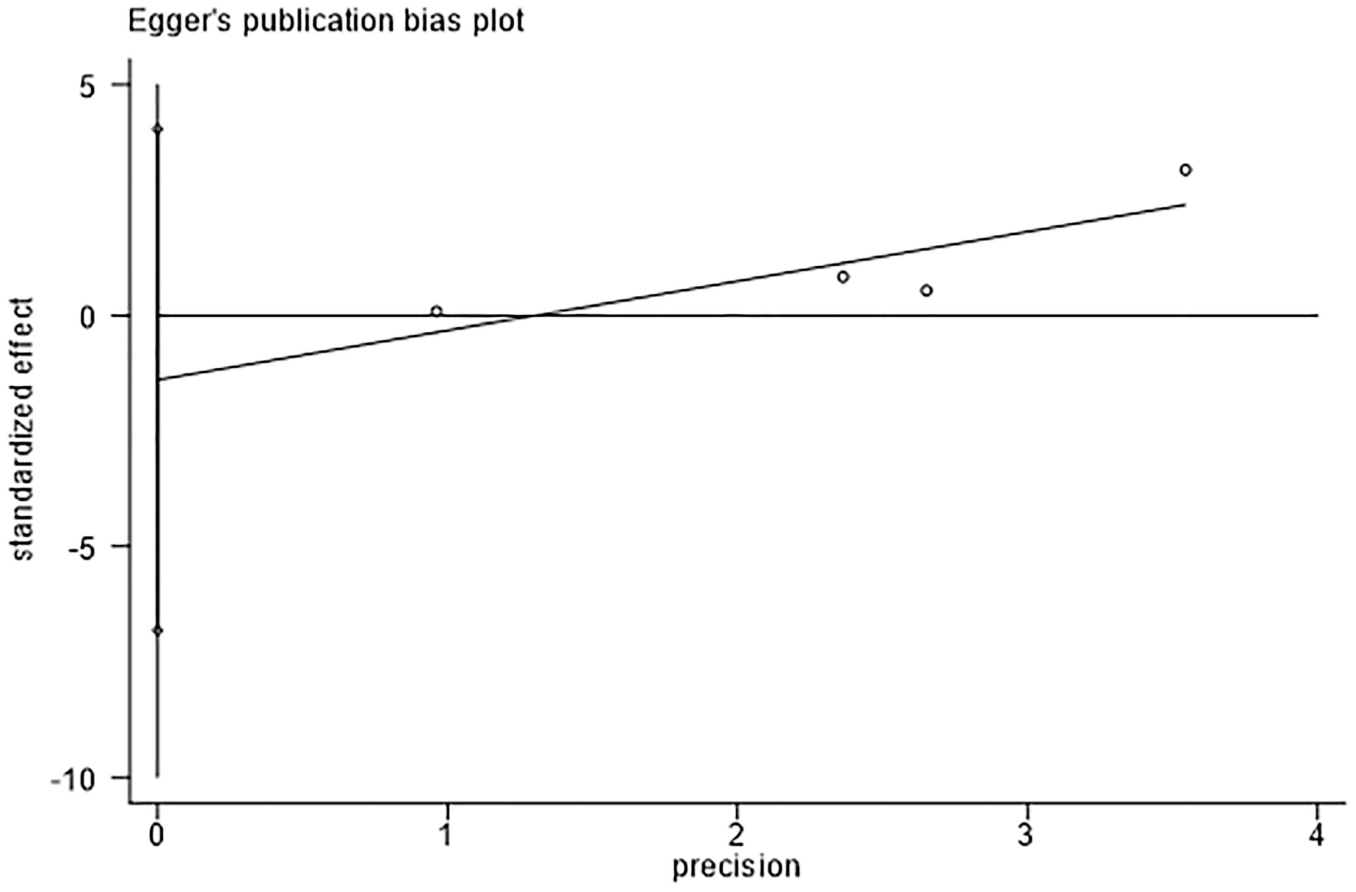
Egger’s test for detection rate of adenoma indicates that there was no substantial publication bias.

The assessment of publication bias using Egger’s test revealed no evidence of bias in the detection rates of adenomas. (Egger’s test, *P* = 0.384, **Figure 5**).

### 3.7 Trial sequential analysis

Trial Sequential Analysis (TSA) addresses the limitations inherent in traditional meta-analysis, rendering the results of statistical analysis more robus. Additionally, TSA can estimate the Required Information Size (RIS) that a meta-analysis needs to achieve a stable conclusion, thereby offering a termination criterion for the sample size of clinical trials (21). In this study, TSA was applied to four studies focusing on the incidence of new adenomas, with type 1 error set at 0.05, power at 0.80, and the RIS designated as the requisite sample size for the meta-analysis (24).

**Figure 6:**
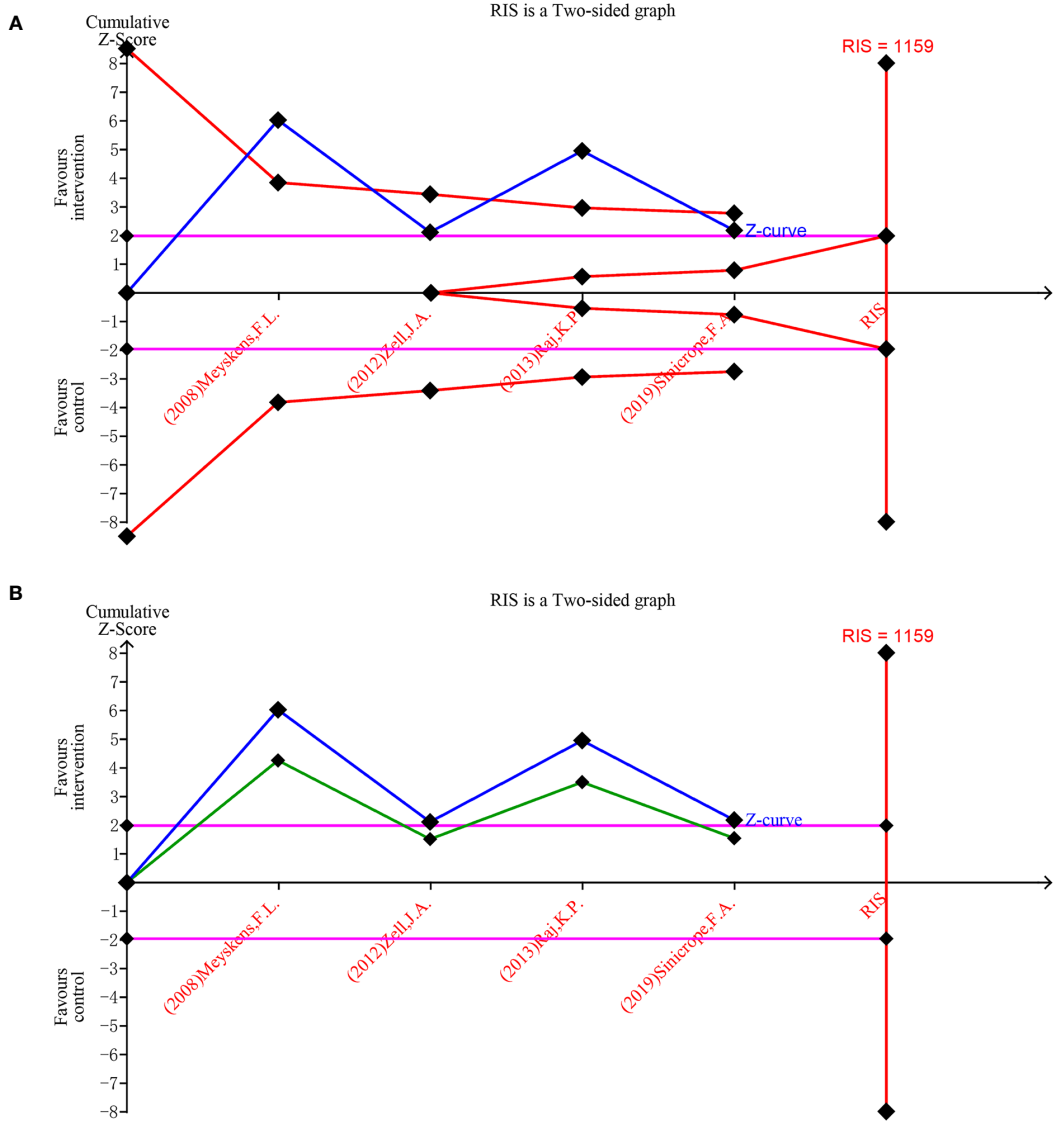
Forest plot for the detection rate of recurrent adenoma in patients with colorectal cancer compared with DFMO combination therapy and placebo. **(A)** Random effects model of the TSA of detection rate of recurrent adenoma. A diversity-adjusted information size of 1159 participants was calculated on the basis of using α=5% (two-sided), β=20% (power 80%), and I2 = 82%. The solid blue line represents the cumulative Z-curve, which crossed the futility boundary (solid red line); **(B)** Penalty statistic analysis of the incidence of recurrent adenoma with DFMO combination. This conclusion is further confirmed by the fact that the penalty curve (solid green line) exceeds the traditional threshold value after inclusion of the first study (solid purple line, Z = 1.96).

The trial sequential analysis for DFMO combination therapy regarding the incidence of recurrent adenomas revealed that the estimated required information size was 1159, while the organized information size fell short of this value, standing at 677. The findings indicate that the Z-curve concurrently crosses both the traditional boundary and the TSA boundary. This suggests that, even though the accumulated information size has yet to meet the anticipated value, no additional trials are necessary, and early confirmation can be achieved. This denotes that the current data is ample to ascertain the efficacy of DFMO combination therapy in addressing the incidence of recurrent colorectal adenomas, as illustrated in **Figure 6A**. The curve surpassing the traditional boundary post-penalty further substantiates this conclusion, as depicted in **Figure 6B**.

### 3.8 GRADE evidence evaluation

The GRADE evidence profile for the primary outcome is shown in **Table 2**. The GRADE working group graded the evidence for recurrent adenoma detection rate as intermediate and the evidence for disease progression as very low. Indirectness and publication bias were not detected.

**Table 2:** GRADE evidence profiles.

Studies	Risk of bias	Inconsistency	Indirectness	Imprecision	Publication bias	Relative effect (95% CI)	No of Participants (studies)	Overall certainty of the evidence
Disease progression	-1	-1	0	-2	0	RR 0.52 (0.14 to 1.86)	221(2 studies)	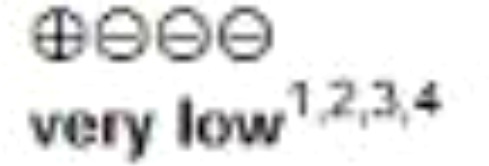
The detection rate of adenoma	-1	0	0	0	0	RR 0.33 (0.12 to 0.9)	677 (4 studies)	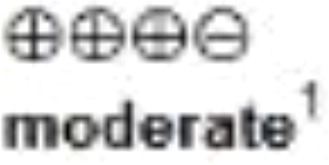

^1^Random, hidden, or blind methods have drawbacks.

^2^50% < I²< 75% after the combination of the included data.

^3^95% CI crossed the invalid line.

^4^Sample size was small (continuity variable < 400, dichotomies < 300).

## 4 Discussion

This study comprehensively and systematically reviewed the literature on the efficacy of DFMO chemoprevention in a high-risk population for CRC. The RCT data shows that DFMO combination significantly reduced the incidence of adenomas in patients with recurrent colorectal neoplasms, quality of assessment evidence based on GRADE methodology “medium”. There was no difference in the control of disease progression in familial adenomatous polyposis with DFMO combination therapy. This study demonstrated robust results through sensitivity analysis.

The combined outcome of the two studies demonstrated no difference in the control of the progression of disease in familial adenomatous polyposis between the DFMO combination with sulindac. However, one study confirmed (25) that sulindac was effective in delaying colonic polyposis progression in patients with familial adenomatous polyposis. The reason for the non-significant combined result may be that the sulindac-treated group (9) showed a lower observed event rate of disease progression than the expected 70%, thus affecting the final results. Future investigation is expected to authenticate the contribution of DFMO in combination or alone in controlling the progression of disease in familial adenomatous polyposis.

The recurrence of adenoma is an important factor that prompts the need for re-treatment of CRC. The current dilemma of a high recurrence rate after resection of colorectal adenoma and early CRC lesions exists. Existing studies suggest that the detection rate of colorectal adenoma by repeat colonoscopy in the first year after surgery is as high as 36%-61% (26–29). Hence, there is an urgent need to explore effective programs to prevent the recurrence of CRC. As demonstrated by this study, the combined results of four studies suggest that the use of DFMO in combination with NSAIDs is efficacious in lowering the incidence of new adenomas in patients with previous CRC. Subgroup analysis exhibited that DFMO combined with aspirin did not significantly reduce adenoma recurrence, but the combination of drugs remarkably decreased the number of abnormal rectal crypt foci, consistent with chemoprevention, according to the results of the study (11). Janakiram, N. B. et al. (30) used low-dose DFMO in combination with rosuvastain in 8-week-old CRC rats and found that the combination therapy was effective in inhibiting the proliferation rate of CRC by 76%. Additionally, Patlolla, J. M. R. et al. (31) used DFMO in combination with sulindac in 7-week-old CRC rats. They showed that the combination therapy effectively reduced the incidence of colorectal adenomas by 42%, especially for adenocarcinomas > 0.5 mm. In addition, the combination therapy also significantly enhanced the expression of p21WAF1/CIP1, caspase 3 cleavage, and down-regulated the expression of bcl-xL, bcl-2, and surviving in rat colon tumor tissues. The sequential analysis shows that although the sample size is not reached, the reliability of the data is satisfied.

Increased ODC activity accompanied by tumor transformation, and elevated polyamine concentrations further promoted tumor development (32). Therefore, several strategies to reduce intracellular polyamine levels have been investigated to study their efficacy in cancer prevention. One successful strategy is the use of DFMO in combination with NSAIDs to reduce polyamine levels to achieve tumor suppressive effects. DFMO reduces the number and size of colonic adenomas and significantly decreases tumor cell proliferation by inhibiting ODC and promoting apoptosis pathways to lower polyamine levels (31). NSAIDs combat CRC by promoting polyamine export through the activation of spermidine/Spermine -N1-acetyltransferase (33–35). Polyamines are elevated in adenomatous colon polyps and CRC compared with normal mucosa, and related studies have shown that DFMO combined with sulindac significantly reduces polyamine production in rectal mucosa (36–38) and inhibits the growth and activity of human CRC cells (39), with good synergistic effects (40). In animal models of colon cancer, DFMO, promoted a significant reduction in intestinal tumors compared with DFMO alone (11, 41). The low-dose combination therapy contributed to a significant downregulation of the inflammatory markers IL-6, stat3, and COX-2, along with the proliferation markers β-catenin and cyclin D1, in rat tumors compared with low-dose resulvastatin or DFMO alone (30). Thus, this combination of DFMO and sulindac or aspirin has been identified as an effective inhibitor for the prevention of CRC (42).

It is important to note that DFMO is labeled as pregnancy category C and should be used with caution in pregnant women because the drug has been found to reduce fetal weight and may cause skeletal variation (43). While evidence (44–47) points to DFMO inducing clinical ototoxicity, evident through symptoms like tinnitus, hearing impairment, and vertigo (32, 48). concurrent research underscores that this ototoxicity is dose-responsive. Notably, these symptoms tend to subside within three months of discontinuing the medication (32). Additionally, the usual therapeutic dose (500 mg/m^2^ per day) did not cause clinically detectable ototoxicity (49–51). In addition, compared with many other oncology treatments, DFMO is administered in an oral form, which is easy to take and can improve the quality of patient survival. The chemical synthesis of the drug is mature and stable for long-term storage and is relatively cheaper, which can benefit low-income patients (51). Good compliance is also an important reason for the recommendation of this drug, as previous phase III clinical trials have shown that patients have a 95% compliance rate with this drug (49). Therefore, DFMO, in combination with NSAIDs, is recommended as a combination therapy for the chemoprevention of CRC.

This study was evaluated by the Grade evidence, which showed moderate evidence that the DFMO combination was effective in reducing the incidence of adenomas in patients with previous colorectal neoplasms and could provide some reference for clinical practitioners. The other two outcome indicator had extremely low outcome indicator evidence, most notably related to imprecision, i.e., due to small sample size and wide confidence intervals. It is anticipated that future research endeavors focusing on chemoprevention studies involving DFMO in combination with NSAIDs, will have the opportunity to delve deeper into determining the optimal dosage and regimen for preventing colorectal tumors in high-risk patient groups. These findings may serve as valuable references for clinical practitioners.

### 4.1 Strengths and limitations

The strengths of this meta-analysis lie in the conformity with the PRISMA statement and the certainty of applying the GRADE approach to evaluate the evidence. We verified the reliability of our findings through Trial Sequential Analysis (TSA), thereby affirming the authenticity of the results prematurely and circumventing the unnecessary expenditure of clinical resources. Our meta-analysis has some shortcomings that may influence the interpretation of the outcome. First, it is difficult to completely rule out the presence of publication bias, as this meta-analysis included only six trials. Second, data limitations prevented further subgroup analyses to explore the effects of different doses and follow-up period of drugs on outcome indicators.

## 5 Conclusions

In conclusion, this meta-analysis indicates that the presently available randomized controlled trials are adequate to conclusively ascertain the effectiveness of DFMO combination therapy, particularly in reducing the incidence of recurrent adenomas. Based on the assessment of evidence quality, the quality of evidence from previous randomized controlled trials is deemed “moderate,” making it a viable reference.

## Data availability statement

The original contributions presented in the study are included in the article/supplementary material. Further inquiries can be directed to the corresponding authors.

## Author contributions

LY: Conceptualization, Data curation, Methodology, Resources, Visualization, Writing – original draft, Writing – review & editing. YW: Writing – original draft, Writing – review & editing. SH: Conceptualization, Data curation, Software, Writing – original draft, Writing – review & editing. XW: Formal Analysis, Investigation, Software, Supervision, Writing – review & editing, Writing – original draft.
